# Disparities in Non-Small Cell Lung Cancer (NSCLC) by Age, Sex, and Race: A Systematic Review and Meta-Analysis of Immune Checkpoint Inhibitor (ICI) Trials

**DOI:** 10.3390/cancers18010128

**Published:** 2025-12-30

**Authors:** Maxim Yaskolko, Christopher Liu, Alexander Barsouk, Jonathan H. Sussman, Adam A. Barsouk

**Affiliations:** 1 Perelman School of Medicine, University of Pennsylvania, 3400 Civic Center Boulevard, Philadelphia, PA 19104, USA; maxim.yaskolko@pennmedicine.upenn.edu (M.Y.);; 2 Irvine School of Medicine, University of California, 1001 Health Sciences Rd, Irvine, CA 92697, USA; christzl@hs.uci.edu; 3 Allegheny Health Network, Pittsburgh, PA 15212, USA; 4 Graduate Group in Genomics and Computational Biology, Perelman School of Medicine, University of Pennsylvania, Philadelphia, PA 19104, USA; 5 Hospital of the University of Pennsylvania, Abramson Cancer Center, Philadelphia, PA 19104, USA

**Keywords:** NSCLC, immunotherapy, disparities, age, sex, race, survival, PD-1, PD-L1, CTLA-4

## Abstract

**Background:** While immune checkpoint inhibitors (ICIs) have improved survival in mNSCLC, outcomes may be disparate by age, race, and sex. However, given the limited diversity of trial populations, data are limited. **Methods:** A systematic review and meta-analysis were conducted of phase III prospective trials of ICIs in mNSCLC initiated after 2015, identified from PubMed and ClinicalTrials.gov in September 2025. Trials that did not report overall survival by race, sex, or age distribution were excluded. Random-effects meta-analyses were used to pool ratios of hazard ratios (RHRs) for overall survival to assess treatment-by-subgroup interactions by sex (men vs. women), race (white vs. Asian), and age (<65 vs. ≥65). Random-effects meta-analyses of odds ratios (ORs) for death were also performed for each subgroup in the investigational treatment arm and across all patients. Heterogeneity across trials was evaluated by Cochran’s Q test and I^2^ statistics. Publication bias was assessed by Egger’s tests and funnel plots. **Results:** A total of 21 trials comprising 10,950 patients were included in the meta-analysis. Women have a non-significantly smaller overall survival benefit with the investigational treatment than with control compared with men (RHR 0.91; *p* = 0.17). On investigational agents, white patients had higher odds of death (OR 1.76; *p* = 0.0496) compared to Asian patients. Similarly, white patients had higher odds of death across both treatment arms compared to Asian patients (OR 2.35; *p* < 0.001). No trials reported subgroup analysis for Black patients due to small sample sizes. Patients ≥65 years old have a non-significantly smaller overall survival benefit with investigational agents compared with control (RHR 0.92; *p* = 0.19). **Discussion:** This study did not identify significant differences in overall survival benefit on the investigational treatment across race, sex, or age subgroups. Still, it remains unclear whether women and patients ≥65 years old derive less survival benefit from ICIs than men and younger patients. Asian patients had significantly greater survival than white patients on investigational therapies and all therapies, while Black patients were underrepresented in trials. Our results highlight the need for more representative trial populations and standardized reporting of subgroup analysis to ensure equitable benefit and evaluation of ICIs in mNSCLC treatment. Our meta-analysis was limited by inconsistent data reporting across subgroups and by the lack of time-to-event survival data within subgroups.

## Simple Summary

We conducted a meta-analysis of all immunotherapy (PD-1, PD-L1, CTLA-4) trials in non-small cell lung cancer (NSCLC) for survival disparities by race, sex, and age. We found that Asian patients had higher rates of survival compared to White patients on immunotherapy and overall. Black patients were underrepresented in the trials. We did not find any difference in survival by sex or age. Furhter research and greater diversity in NSCLC trials is required to ensure optimal outcomes for all patient populations.

## 1. Introduction

Non-small cell lung cancer (NSCLC) remains the leading cause of cancer mortality worldwide, with immune checkpoint inhibitors (ICIs) revolutionizing treatment and improving survival in advanced disease [1,2]. PD-1 and PD-L1 inhibitors pembrolizumab, cemiplimab, atezolizumab, nivolumab (+/−) ipilimumab (a CTLA-4 inhibitor), and durvalumab + tremelimumab (CTLA-4 inhibitor) have all been FDA approved for wild-type 1L mNSCLC, largely in combination with platinum chemotherapy; pembrolizumab, cemipilimab, and atezolizumab are also approved as monotherapies for patients with PDL1 TPS expression >50%. Novel therapeutics in development include PD1xVEGF bispecifics like ivonescimab, antibody drug conjugates like datopotomab deruxtecan and sacituzimab govetecan, and nanotechnology enabled sensitizers. Despite these advances, disparities in clinical outcomes and trial representation persist across age, sex, and racial groups [3].

Older adults are historically underrepresented in ICI trials, yet available evidence suggests comparable efficacy and toxicity profiles to younger patients, though some studies indicate attenuated benefit in the oldest cohorts [4,5,6,7]. Disparities in ICI efficacy by sex have been explored, with most meta-analyses reporting similar survival benefits for men and women. However, some data suggest women may derive greater benefit from combination regimens, and tumor mutational burden may modulate sex disparities [8,9,10,11,12]. Racial disparities are evident in both trial enrollment and real-world access to ICIs. Black and Hispanic patients have remained underrepresented in prospective NSCLC trials in the US [13], limiting conclusions on disparities. While limited meta-analyses indicate similar survival benefits for White and Asian patients [14,15,16], some meta-analyses across tumor types have suggested Asian patients may have superior survival over white patients [17,18].

Prior studies have reported inconsistent findings regarding disparities in enrollment or treatment efficacy by age, sex, and race among NSCLC patients treated with ICI therapy. This comprehensive meta-analysis of phase III clinical trials, those best designed to evaluate efficacy, aims to systematically identify disparities in enrollment and reporting, and to clarify the impact of age, sex, and race on ICI efficacy in NSCLC. These findings can inform clinical decision-making and guide future trial design toward more equitable cancer care.

## 2. Methods

### 2.1. Search Strategy

We conducted a systematic review of completed prospective phase III trials in mNSCLC evaluating ICIs (pembrolizumab, atezolizumab, nivolumab +/− ipilimumab, durvalumab +/− tremelimumab) initiated after 2015 using PubMed and ClinicalTrials.gov. This approach selects trials of the highest methodological quality and minimal bias that are most likely to provide complete and relevant evidence in the current therapeutic landscape. The analysis was conducted in September 2025. We used the following search string to identify 60 trials: AREA[ConditionSearch](Non Small Cell Lung Cancer) AND AREA[StartDate] RANGE [1 January 2015, MAX] AND AREA[OverallStatus](COMPLETED) AND AREA[Phase](PHASE3) AND AREA[StudyType](INTERVENTIONAL) AND (AREA[HasResults] true). Two duplicate studies were excluded, and 10 studies that did not investigate ICIs were excluded.

### 2.2. Inclusion Criteria

Trials were evaluated for inclusion of baseline demographics—age, sex, and race—and for subgroup analyses of overall survival (OS) by age, sex, and race in the published manuscript. Two authors independently conducted the screening, and for any paper identified by one author but not the other, a third author was consulted to determine if the identified report met the inclusion criteria, which was then taken as the final decision.

### 2.3. Statistical Analysis

One author performed all data extraction, which a second author independently verified. Baseline demographic data for age, sex, and race were extracted from each trial. The primary outcome was the difference in OS benefit between subgroups, estimated by analyzing treatment-by-subgroup interactions using the ratio of hazard ratios (RHRs) for death, calculated from reported hazard ratios. Meta-analyses of these interaction effects were conducted for men vs. women, white vs. Asian, and for patients aged < 65 vs. ≥65. The secondary outcome was the difference in odds of death between subgroups, estimated by calculating odds ratios (ORs) for death events in both investigational and control arms. Meta-analyses of ORs for death were performed for the above subgroup comparisons in the investigational treatment arm and across all study arms. The primary outcome assesses relative treatment effect modification, while the secondary outcome evaluates absolute differences in mortality. For each meta-analysis, only studies with complete data for the relevant subgroup analysis were included. Random-effects models were used to pool results across studies in all meta-analyses to account for the inherent heterogeneity arising from differences in trial design, treatments, aims, and populations. Heterogeneity was also evaluated by Cochran’s Q test and I^2^ values. Significant heterogeneity was further investigated with leave-one-out sensitivity analysis. *p* values < 0.05 were considered significant. R v4.4.0 was used for statistical analysis (Foundation for Statistical Computing, Vienna, Austria). The meta for R package v4.5.2 was used for all meta-analyses. This systematic review followed the PRISMA 2020 guidelines and was not prospectively registered [19].

### 2.4. Bias Assessment

Publication bias was evaluated using Egger’s tests and funnel plots. Risk of bias was assessed using the Cochrane risk of bias tool 2, which rates each study on the following domains: randomization, deviations from intended interventions, missing outcome data, measurement of outcome, and selection of reported results.

## 3. Results

**Figure 1:**
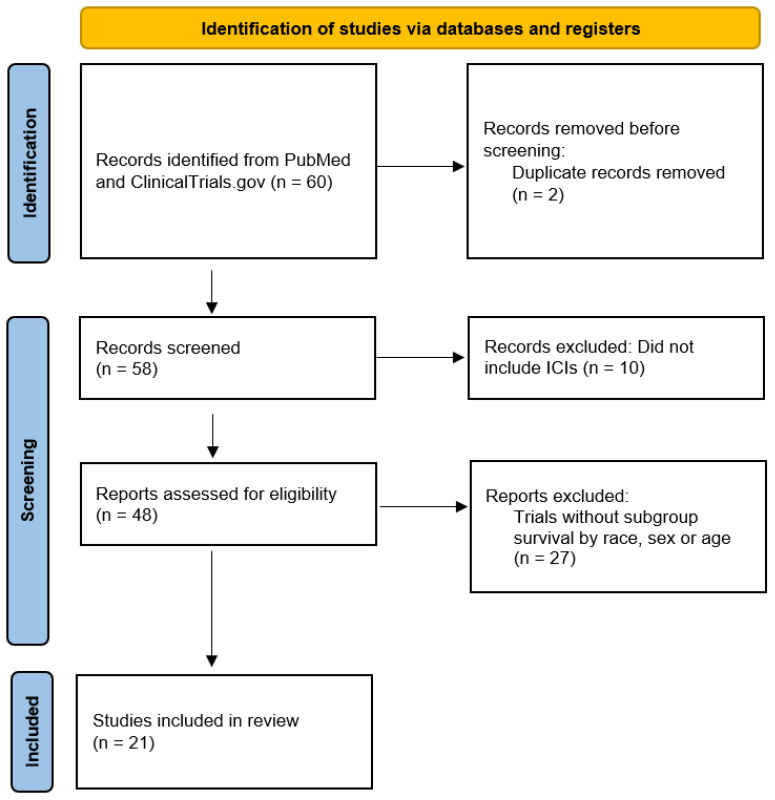
Review Methodology PRISMA diagram. A systematic review of PubMed and ClinicalTrials.gov was performed in September 2025. The following search string was used to search ClinicalTrials.gov: AREA[ConditionSearch](Non Small Cell Lung Cancer) AND AREA[StartDate] RANGE [1 January 2015, MAX] AND AREA[OverallStatus](COMPLETED) AND AREA[Phase](PHASE3) AND AREA[StudyType](INTERVENTIONAL) AND (AREA[HasResults] true). Trials without ICIs as the investigational agent were excluded. Remaining trials were then separated based on whether subgroup survival by race, sex, or age was reported.

Our literature search identified 21 distinct trials (Figure 1). Two studies were multi-arm, yielding 23 total pairwise comparisons between investigational and control arms. Table 1 presents key characteristics of the trials and the significance of the OS benefit with the investigational treatment versus control across sex, race, and age subgroups. No trials were judged to be at high risk for bias. Some concerns were identified in all trials for bias due to the selection of reported results from limited demographic reporting or exploratory subgroup analyses (n = 23).

### 3.1. Sex

**Figure 2:**
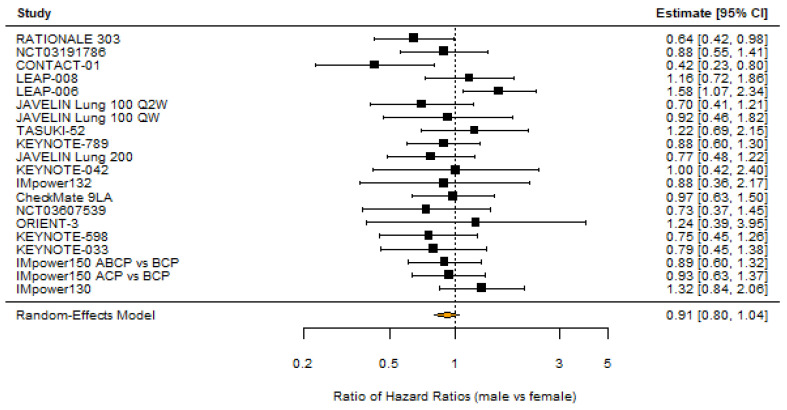
Forest plot of OS benefit in investigational vs. control arm (male vs. female patients) [21,22,23,24,25,26,27,28,29,30,31,32,33,34,35,36,37,38,39,40].

**Figure 3:**
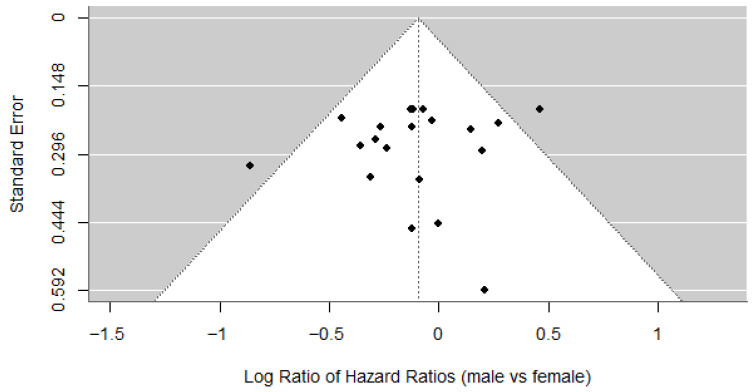
Funnel plot of log ratio of hazard ratios (male vs. female patients).

All trials (n = 10,950) reported sex distribution and OS subgroup analysis by sex. 68.5% of patients were men. 11/23 (47.8%) comparisons between investigational and control arms found that men had a significant overall survival benefit over control, while only 1/23 (4.5%) found that women had a significant overall survival benefit over control. A random-effects meta-analysis of the 20 comparisons that reported overall survival hazard ratios by sex suggested that women had a non-significantly smaller OS benefit with the investigational treatment compared to control (RHR 0.91 [95% CI: 0.80–1.04]; *p* = 0.17; Figure 2). Heterogeneity was low and not significant (I^2^ = 25.4%; *p* = 0.21), and there was no evidence of publication bias based on Egger’s test (*p* = 0.45) and a symmetrical funnel plot (Figure 3). Random-effects meta-analysis of the nine studies that reported deaths by sex in the investigational arms found that men did not have lower odds of death in the investigational arms (OR 0.98 [95% CI: 0.73–1.32]; *p* = 0.89).

### 3.2. Race

**Figure 4:**
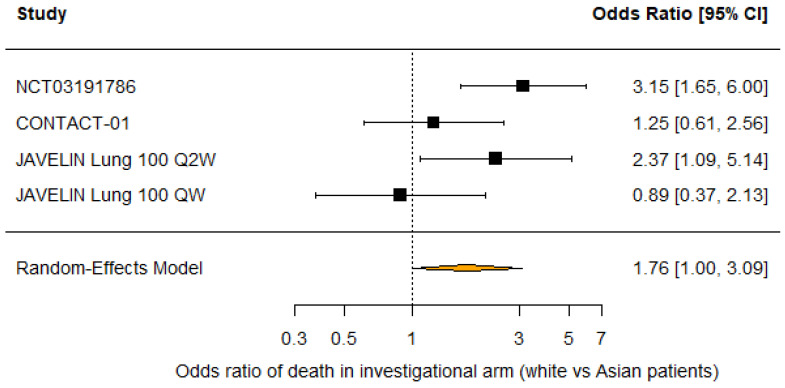
Forest plot of odds ratios of death in the investigational arm (white vs. Asian patients) [22,23,26].

**Figure 5:**
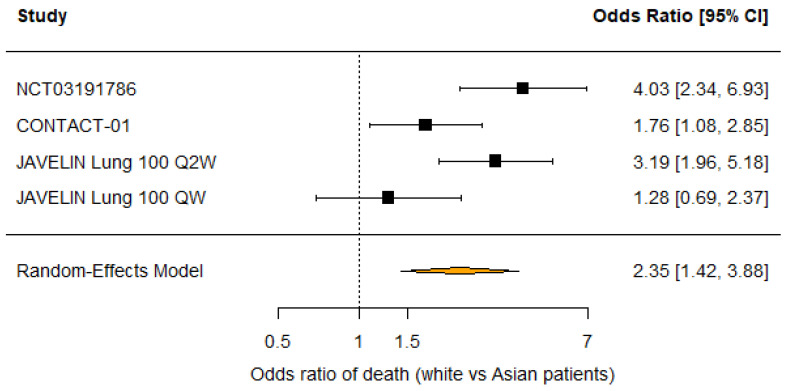
Forest plot of odds ratios of death in all trial patients (white vs. Asian patients) [22,23,26].

**Figure 6:**
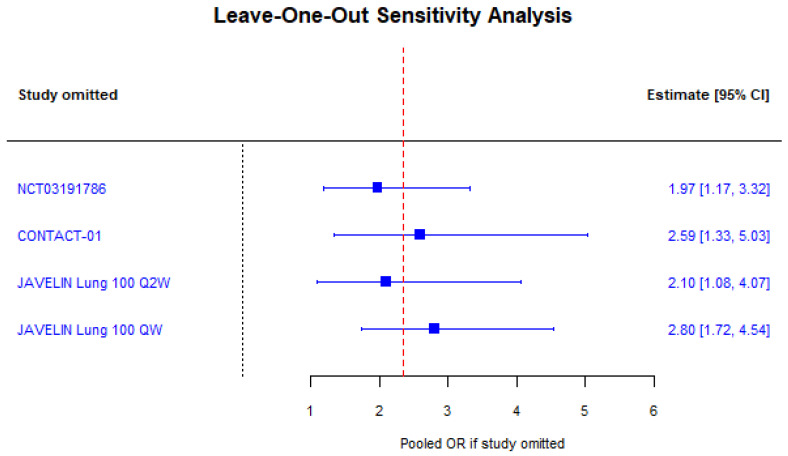
Leave-one-out sensitivity analysis forest plot of pooled odds ratios of death across all trial patients (white vs. Asian patients) [22,23,26].

13/21 (61.9%) trials analyzed reported race distribution with 59.2% white, 34.8% Asian, 2.7% Native American or Alaskan Native patients, and only 1.5% Black patients. 7/21 (33.3%) trials reported OS subgroup analysis by race. 2/8 (25%) comparisons between investigational and control arms found that white patients had a significant overall survival benefit from the investigational treatment, while 1/6 (16.7%) comparisons found that Asian patients had a significant survival benefit. No trials reported subgroup analysis for Black patients due to small sample sizes. A random-effects meta-analysis of the five comparisons that reported overall survival hazard ratios for white and Asian patients suggested no difference in OS benefit with the investigational treatment compared to control (RHR 0.95 [95% CI: 0.73 −1.24]; *p* = 0.72). However, random-effects meta-analysis of the four comparisons that reported deaths by race in the investigational arm found that white patients had higher odds of death compared to Asian patients (OR 1.76 [95% CI: 1.00–3.09]; *p* = 0.0496; Figure 4). Similarly, white patients had higher odds of death across both treatment arms compared to Asian patients (OR 2.35 [95% CI: 1.42–3.88]; *p* < 0.001; Figure 5). Moderate heterogeneity in both analyses (I^2^ = 56.1%; *p* = 0.08 and I^2^ = 72.2%; *p* = 0.02) should be interpreted with caution as only four studies were included in each analysis. Furthermore, leave-one-out sensitivity analysis demonstrated that excluding any study did not significantly alter the pooled odds ratio of death among all patients, indicating that our results are robust (Figure 6). There was no evidence of publication bias by Egger’s test (*p* = 0.25 and *p* = 0.64).

### 3.3. Age

**Figure 7:**
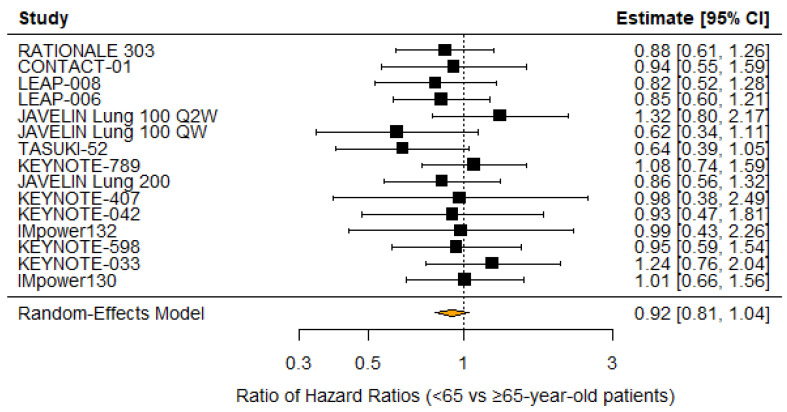
Forest plot of OS benefit in investigational vs. control arm (<65 vs. ≥65-year-old patients) [21,23,24,25,26,27,28,29,30,31,33,37,38,40].

18/21 (85.7%) trials reported distribution by age ≥ 65 years vs. <65 years old, with 55.9% of patients <65 years old. 20 comparisons between investigational and control arms reported OS subgroup analysis by the <65 cutoff, while 17 reported death by the ≥65 cutoff. 7/20 (35%) found that patients <65 years old had a significant OS benefit with the investigational treatment, while 4/17 (23.5%) found that patients ≥65 years old had a significant OS benefit. A random-effects meta-analysis of the 15 comparisons that reported overall survival hazard ratios by age suggested that patients ≥65 years old have a non-significantly smaller OS benefit (RHR 0.92 [95% CI: 0.81–1.04]; *p* = 0.19; Figure 7). Random-effects meta-analysis of the 13 studies that reported deaths by age in the investigational arms found that men did not have lower odds of death in the investigational arms (OR 0.94 [95% CI: 0.77–1.15]; *p* = 0.54). No heterogeneity (I^2^ = 0; *p* = 0.84) or publication bias was detected by Egger’s test (*p* = 1.00).

## 4. Discussion

### 4.1. Disparity in Survival by Sex

All but one study failed to demonstrate that women benefited from investigational treatment compared with control. Still, our meta-analyses suggested that men and women had similar OS, and there was no significant difference in the benefit from investigational treatments, although the point estimate was a lower benefit for women (RHR 0.91, *p* = 0.17). Increased inclusion of women is needed to further explore this potential disparity. These results are in keeping with the existing literature.

Large meta-analyses and real-world studies consistently demonstrate that ICIs improve overall survival (OS) and progression-free survival (PFS) in mNSCLC for both sexes compared to chemotherapy, with most studies reporting no statistically significant difference in the magnitude of benefit between men and women [10,11]. However, some studies and meta-analyses suggest that men may derive greater benefit from ICI monotherapy, while women may benefit more from ICI-chemotherapy combinations [41,42]. For example, Conforti et al. found that women had a significantly greater survival benefit from ICI plus chemotherapy compared to men, whereas men benefited more from ICI monotherapy [9]. Similarly, Yu et al. reported that female patients had markedly better outcomes with PD-1 blockade plus chemotherapy than males, despite lower tumor mutational burden (TMB) in females [43].

Biological factors such as immune microenvironment, cytokine profiles, and TMB may underlie these differences. Females tend to have stronger humoral and cell-mediated immune responses, and sex-specific cytokine signatures (e.g., CXCL10, CCL5) have been linked to differential responses to anti-PD-1 therapy [12,44]. High TMB appears to equalize ICI efficacy between sexes, while low TMB may be associated with poorer outcomes in males, although the etiology remains unclear [13]. Real-world data and registry studies also show that females with advanced NSCLC generally have longer survival than males, even after adjusting for confounders, but the reasons remain incompletely understood, and we did not observe this effect in our analyses [13,44,45].

### 4.2. Disparity in Survival by Race

We found that Asian patients had decreased odds of death compared to white patients, both in the investigational arm and among all trial patients. While the *p* value for the comparison in the investigational arm was close to 0.05, indicating a higher chance that the difference in odds of death is due to random chance, the stronger effect size and *p* value among all trial patients support our conclusion that Asian patients have decreased odds of death compared to white patients. We also found significant underrepresentation of Black patients, which is in keeping with the literature. No studies reported subgroup survival analysis of Black or Native American or Alaskan Native patients. Racial disparities in survival for patients with non-small cell lung cancer (NSCLC) treated with ICIs are primarily driven by differences in access to therapy, but among those who receive ICIs, survival outcomes are generally similar across racial groups, with some studies suggesting equal or even superior outcomes for certain minorities.

Many Asian patients in our meta-analysis came from studies of predominantly or exclusively Asian patient populations that cited the need to explore potential racial differences in ICI response as a rationale for their population selection. This approach of intentional inclusion may be valuable in increasing the representation of Black and other minority groups in clinical trials.

Multiple large registry and database studies show that Black, Hispanic, and Asian patients are less likely to receive ICI therapy compared to White patients, even after adjusting for clinical and socioeconomic factors [46,47]. This disparity in access is a major contributor to overall survival differences at the population level. When minority patients do receive ICIs, most studies report no decrease in overall survival (OS) or progression-free survival (PFS) compared to White patients [48,49,50]. For example, a National Cancer Database analysis found that non-Hispanic Black patients had a lower risk of death than non-Hispanic White patients after adjusting for confounders (HR 0.85) [4,5,6,7]. Similarly, real-world and clinical trial data show comparable efficacy and safety of ICIs in Black and white patients [4]. In one study, Asian patients in the US were reported to exhibit longer survival than white or Black patients with mNSCLC, despite lower rates of receipt of ICIs [51]. Similarly, meta-analyses of ICI use across solid tumors [17], and in bladder cancer [18], have reported longer survival for Asian than White patients when treated with ICIs. While differences in tumor mutation profiles and PD-L1 expression may contribute to outcome variation, these do not fully explain survival disparities and are likely less influential than access and social determinants [52].

### 4.3. Disparity in Survival by Age

We found older patients ≥65 years old were underrepresented in mNSCLC clinical trials and there was no significant difference in OS benefit from investigational treatments compared with patients <65 years old, although the point estimate indicated a lower benefit for older patients (RHR 0.92, *p* = 0.19). Patients ≥65 also did not have increased odds of death. ICIs have improved survival for patients with advanced NSCLC across all age groups, but disparities persist in the magnitude of benefit for older adults. Subgroup analyses and meta-analyses consistently demonstrate that patients ≥65 years old experience similar overall survival (OS) and progression-free survival (PFS) benefits from ICIs compared to younger patients, with hazard ratios for OS typically ranging from 0.74 to 0.81 for older adults, indicating a meaningful reduction in risk of death compared to chemotherapy [4,5,6]. However, patients aged ≥75 or ≥80 years may experience attenuated survival gains. Several meta-analyses and real-world studies report that while ICIs remain effective in these age groups, the OS benefit is less pronounced, and some studies fail to show statistically significant improvement over chemotherapy for patients ≥75 years. For example, pooled analyses show HRs for OS approaching 1.0 in patients ≥75 years, and real-world data indicate shorter median OS in patients ≥80 years, often attributed to lower rates of second-line therapy and increased comorbidities [1,52,53].

Performance status and comorbidities are critical determinants of ICI benefit in older adults. 2025 guidelines from the National Comprehensive Cancer Network (NCCN) recommend that older patients with good performance status (Eastern Cooperative Oncology Group 0–2) and manageable comorbidities should receive standard ICI-based regimens, while frail patients or those with poor performance status may benefit from single-agent ICI or supportive care [6]. The IPSOS and CheckMate 817 trials support using single-agent atezolizumab or dual checkpoint inhibition in platinum-ineligible or frail older patients, with improved survival and manageable toxicity [20,54].

Toxicity profiles are generally similar across age groups, but older patients may be at higher risk for severe immune-related adverse events (irAEs). While some studies suggest younger patients are more likely to experience irAEs, others report higher rates of severe irAEs in those over 75, though overall rates remain acceptable and do not preclude ICI use based on age alone [6].

Immunosenescence and distinct tumor microenvironment features in older adults may influence ICI response, but do not preclude efficacy. Studies of patients ≥80 years with PD-L1-high NSCLC show similar objective response rates and PFS to younger patients, though OS may be shorter due to lower use of subsequent therapy and increased comorbidities [55,56,57].

### 4.4. Limitations

Our meta-analysis was limited by the lack of diversity among trial populations, particularly with poor inclusion of Black patients, women, and older patients (≥65), and a lack of data reporting by subgroups. Multiple analyses of pivotal NSCLC ICI trials show that Black patients typically comprise only 1.9–3% of trial participants, and Hispanic patients about 3–5.9%, despite representing 13% and 18% of the US population, respectively [53,58]. Even when reported, Black and Hispanic patients were underrepresented, causing trials to have insufficient Black patients to include in subgroup analysis. Non-standard age cutoffs (65 vs. 75) also limited data analysis, making it difficult to draw broad conclusions about whether age affects ICI efficacy. Furthermore, since the included studies did not report relevant hazard ratios for subgroup analysis of survival in the investigational treatment, our meta-analyses relied on odds ratios of death to evaluate absolute differences in survival. This approach fails to include time-to-event information. Our work was also restricted to completed trials registered on ClinicalTrials.gov. This approach excludes active studies that may have interim data on subgroup survival. Although only one of our analyses showed significant between-study heterogeneity, the studies we include reflect different populations, treatment combinations, and study designs. Improved trial diversity and standardized subgroup reporting would directly address many of these limitations.

## 5. Conclusions

This analysis demonstrates that prospective phase III trials of ICIs in mNSCLC have persistent underrepresentation of women, older adults, and racial minorities. Additionally, most trials lack subgroup analysis by age, race, or sex, limiting generalizability and our ability to determine how these factors affect treatment efficacy. Among studies reporting overall survival hazard ratios by subgroup, only 1 demonstrated benefit from the investigational treatment in women, compared with 11 in men. Similarly, a greater proportion of studies found that patients <65 years old benefited from the investigational treatment than patients ≥65 years old. This study did not identify significant differences in overall survival benefit on the investigational treatment across race, sex, or age subgroups. Meta-analyses of overall survival hazard ratios identified a non-significantly smaller benefit for women and patients ≥65 years old. Increased reporting of subgroup analyses and greater representation of women and older patients are needed to further evaluate these disparities. Meta-analyses of odds ratios of death revealed that Asian patients had decreased odds of death compared to white patients, in keeping with the existing literature. Black patients were severely underrepresented, making up only 1.5% of patients with reported race. These findings highlight the need for policy initiatives and trial design strategies that facilitate more representative trials and standardized reporting of subgroup analysis, ensuring generalizable and equitable evaluation of ICIs in mNSCLC treatment.

## Figures and Tables

**Table 1:** Characteristics and subgroup OS benefit for investigational treatments of the 21 included trials (NR = not reported).

Source	Trial Name	n	% Male	OS Benefit	% White	% Asian	OS Benefit	% <65	OS Benefit
Mok et al., 2024 [20]	Checkmate 722	294	39.8	Neither	6.1	93.9	Neither	56.8	Neither
Zhou et al., 2023 [21]	RATIONALE 303	805	77.3	Male	17.6	82.4	Both	67.6	Both
Lee et al., 2023 [22]	NCT03191786	453	72.4	Male	73	27.7	Neither	NR	NR
Neal et al., 2024 [23]	CONTACT-01	355	73.5	Male	71.9	28.1	Neither	48.6	Neither
Leighl et al., 2025 [24]	LEAP-008	374	66	Neither	80.7	NR	Neither	48.1	Neither
Herbst et al., 2025 [25]	LEAP-006	748	67	Neither	67.2	NR	Neither	54.4	Neither
Reck et al., 2024 [26]	JAVELIN Lung 100 Q2W38	367	73.6	Neither	71.8	28.2	Neither	54	Neither
	JAVELIN Lung 100 QW	259	74.5	Neither	76.7	23.3	White	54.4	<65
Kim et al., 2023 [27]	TASUKI-52	550	74.7	Neither	NR	NR	NR	44	<65
Yang et al., 2024 [28]	KEYNOTE-789	492	38.4	Neither	NR	NR	NR	55.3	Neither
Barlesi et al., 2018 [29]	JAVELIN Lung 200	529	69.4	Neither	67.2	28.8	NR	52.7	Neither
Cheng et al., 2021 [30]	KEYNOTE-407	125	95.2	Neither	NR	NR	NR	59.2	Both
Wu et al., 2021 [31]	KEYNOTE-042	262	85.5	Male	0	100	NR	65.6	<65
Wu et al., 2019 [32]	CheckMate 078	504	78.8	Male	NR	NR	NR	74.8	≥65
Lu et al., 2023 [33]	IMpower132	163	73	Neither	0	100	NR	68.7	Neither
Paz-Ares et al., 2021 [34]	CheckMate 9LA	719	70.1	Male	NR	NR	NR	49.2	<65
Yang et al., 2020 [35]	NCT03607539	398	76.1	Male	0	100	NR	NR	NR
Shi et al., 2022 [36]	ORIENT-3	280	92.1	Male	NR	NR	NR	NR	NR
Boyer et al., 2021 [37]	KEYNOTE-598	567	69.3	Neither	NR	NR	NR	49.5	Neither
Ren et al., 2023 [38]	KEYNOTE-033	425	75.5	Male	NR	NR	NR	66.1	≥65
Reck et al., 2019 [39]	IMpower150 ABCP vs. BCP	800	59.9	Male	84.1	13.1	NR	55.5	NR
	IMpower150 ACP vs. BCP	802	59.9	Neither	84.7	12	NR	56.3	NR
West et al., [40] 2019	IMpower130	679	58.9	Both	90.1	2.2	NR	50.2	Neither

## Data Availability

No new data were created or analyzed in this study. Analytic code can be made available upon reasonable request.
